# Recycling of Almond By-Products for Intestinal Inflammation: Improvement of Physical-Chemical, Technological and Biological Characteristics of a Dried Almond Skins Extract

**DOI:** 10.3390/pharmaceutics12090884

**Published:** 2020-09-17

**Authors:** Maria Rosaria Lauro, Stefania Marzocco, Shara Francesca Rapa, Teresa Musumeci, Virgilio Giannone, Patrizia Picerno, Rita Patrizia Aquino, Giovanni Puglisi

**Affiliations:** 1 Department of Pharmacy, University of Salerno, Via Giovanni Paolo II, 84084 Fisciano, Italy; smarzocco@unisa.it (S.M.); srapa@unisa.it (S.F.R.); ppicerno@unisa.it (P.P.); aquinorp@unisa.it (R.P.A.); 2 Department of Drug Sciences, University of Catania, Viale A. Doria, 95100 Catania, Italy; tmusumec@unict.it (T.M.); puglisig@unict.it (G.P.); 3 Department of Agricultural and Forest Sciences, University of Palermo, Viale delle Scienze Ed.4, 90128 Palermo, Italy; Giannonevirgilio@gmail.com

**Keywords:** almond extract, cyclodextrin, catechin, solubility studies, complex characterization, intestinal epithelial cells, inflammation, oxidative stress

## Abstract

Background: Almond skins are rich in bioactive compounds that undergo oxidation/degradation phenomena and are poorly soluble in water, reducing in vivo absorption and bioavailability, factors that influence the pharmacological activity of an active product. We developed a dried acetonic almond skins extract/cyclodextrin complex to improve extract solubility, dissolution rate and biological activity. Methods: A lyophilized acetonic almond skin extract was produced. To optimize complex formulation, phase solubility studies and complex characterization (absorption studies, differential scanning calorimetry (DSC), morphology, solubility studies) were performed. To evaluate a possible use in healthy products, tumor necrosis factor-α levels and reactive oxygen species release, as well as cicloxygenase-2 and inducible nitric oxide synthase expression in intestinal epithelial cells, were also evaluated. Results: Phase solubility studies showed a Bs-type profile. A 1:1 dried acetonic almond skins extract/cyclodextrin ratio was able to improve extract water solubility and dissolution rate (100% in 45 min). The UV-Vis spectra of complex revealed a hypsochromic and hyperchromic effect, probably due to a partial inclusion of extract in cyclodextrin cavity through weak bonds, confirmed by DSC and morphology studies. The technological improvement in the extract characteristics also led to better biological activity. In fact, the complex effectively reduces tumor necrosis factor-α levels with respect to the pure extract and significantly inhibits the reactive oxygen species release, even if only at the lower concentration of 5 μg/mL. Conclusion: The complex was able to overcome solubility problems and could be used in inflammatory disease.

## 1. Introduction

The waste and processing by-products of the agri-food industry have recently become a subject of great interest in scientific research. In particular, the trend is to encourage the recycling of by-products to minimize wastes and their disposal costs, employing sophisticate technologies to exploit the bioactive substances of which they are rich in order to produce nutraceutical products with high added value. In 2003, the U.S. Food and Drug Administration (FDA) approved a health claim that consuming 42 g (1.5 oz) of almond daily “as part of a diet low in saturated fat and cholesterol may reduce the risk of heart disease” [1]. European law considers nutraceuticals as food ingredients or medicinal herb if they maintain tissue and organ or induce important modification effects, respectively [2]. Almond co-products are rich in polyphenols and nutritional compounds [3], so they can be used as a functional ingredient in the formulation of health products like dietary supplements. We focused our attention on almond skins. Unfortunately, the compounds like polyphenols responsible for the almond skins’ bioactivity [3,4,5] undergo oxidation/degradation phenomena and are poorly soluble in water. When administered per os, these factors limit the in vivo bioavailability and bioactivity due to the slow dissolution rate in biological fluids. Moreover, almond skins possess “woody” and “earthy” [6] organoleptic characteristics that can reduce patient compliance. Despite this information on the nutritional and healthy qualities of the almond nut or its by-products, nothing is reported on their formulation to be administered as a dietary supplement. To better use these matrices, it would be appropriate to produce a formulated edible extract in order to improve its physicochemical and technological characteristics (solubility, palatability, manageability, stability) and to make it an ideal candidate for the production of health products with potential nutraceutical activity. This could make it possible to further enhance the value of by-products, maintaining a strong relationship with Mediterranean cultivars, which have great outcomes in terms of product healthiness [7]. To achieve this goal, we developed an acetonic almond skins extract (ASE) complex (CD-ASE), starting from a shell co-product of the Sicilian almond industry, and beta-cyclodextrin (CD), to increase the ASE stability, solubility, dissolution rate and biological activity. CD is capable of forming inclusion complexes and acting as an “enhancer” by improving the chemical–physical properties of the host molecule, such as degradation and solubility. In addition, it is able to mask unpleasant flavors, thus also improving the organoleptic characteristics of the active ingredients [8]. CD is inserted in the “GRAS list”, it is already widely used in the food field and to ameliorate the chemical–physical, technological and biological characteristics of herbal extract and natural compounds [9,10]. Moreover, in the literature, it was reported that CD was able to complex (+) catechin, once of the most abundant components of almond skins [11], with optimal results in term of complexation and improvement in technological, biological and stability characteristics [12,13,14,15,16]. On these bases, to optimize the ASE formulation, phase solubility studies were performed. Water solubility and in vitro dissolution tests, according to United States Pharmacopeia (USP 37) parameters, and complex characterization studies (absorption studies, differential scanning calorimetry (DSC), morphology) were performed in order to verify the satisfaction of the applied approach in terms of the transformation of a highly functional dry extract into a “bioactive raw material”, with an improvement in its physicochemical, technological characteristics in terms of drug content. This approach is also part of the growing demand for the production of “green supplements”. Furthermore, considering that polyphenols are able to act on inflammation processes through several mechanisms also related to their antioxidative and radical scavenger properties [17], we also evaluated the radical scavenging activity and the pharmacological potential of these products in an in vitro model of intestinal inflammation, in intestinal epithelial cells (IEC-6).

## 2. Materials and Methods

### 2.1. Materials

Dried Almond Skins (var. Corrente and Tuono mixture) were supplied by “Industria Lavorazione Mandorle Calafiore Paolo dei fratelli Calafiore C. Snc”, Floridia (SR), Italy. Beta-cyclodextrin, (+) catechin (CA), were purchased from Sigma–Aldrich S.r.l. (Milan, Italy). Unless otherwise specified, all reagents and compounds were purchased from Sigma Chemicals Company (Sigma, Milan, Italy). All the other chemicals used in the study were of analytical grade.

### 2.2. Acetonic Almond Skins Extract (ASE) Production and YIELD of the Process (Y)

A total of 100 g of dried almond skins were homogenate by an Ultraturrax (IKA ULTRA-TURRAX T25 digital; IKA^®^-Werke GmbH & Co. KG, Staufen, Germany) for 4 min (run 5) and then were added to AcOOH:H_2_O 8:2 *v*/*v*, to give ASE at 300 rpm stirrer for 1 h. After, ASE was centrifuged (ALC CENTRIFUGE PK 120) (ALC Internation S.r.l., Cologno Monzese, Italy) at 5000 rpm for 10 min three times. The surnatant were collected and the solvents removed by a rotavapor to obtain the dried extracts. Y was calculated by a gravimetric method (CAL-Gibertini (max 110 g, d = 0.1 mg; +15 °C/30 °C) and expressed as percent of weight of final product, with respect to the weight of total materials used. Each analysis was made in triplicate and the results were expressed as average value.

### 2.3. Almond Skins Extract (ASE) Technological Characterization

Pre-Formulation Studies: Since nutraceutical products are orally administered, in order to develop an oral formulation, it is important to carry out pre-formulation studies to evaluate the technological characteristics of the prepared extract. (+) Catechin equivalents (CA) were used as a marker and calculated as reported above. The UV-Vis method was used for all analyses because all instrumentation was online with UV-Vis equipment. UV-Vis method. This assay was performed according to ICH [Q2(R1)] guidelines (% Relative Standard Deviation statistically validation, % RSD). CA concentration was evaluated in a UV-Vis 1601 Shimadzu Europa, Duisburg, Germany, (**λ**max of 280 nm; i1 mm cell) according to Lambert–Beer Law (USP 37).

E^1%^_1 cm_ × c × l

where E^1%^_1 cm_: absorbance of 1 g/100 mL (1% *w*/*v*) solution in 1 cm cell; c: concentration of the solution (g/100 mL); l: cell pathlength held sample.

Validation method: the active concentration was calculated using the standard calibration curve.

Intra-day and inter-day precision: The same concentrations of CA (10 mg/L) were analyzed for 6 h and 3 days, respectively.

Linearity: The proportionality between absorbance and concentration was verified at three concentration levels (room temperature; range from 2.5 to 150.0 mgL^−1^; y = 151.47x − 0.754, R^2^ = 0.997, where y is the absorbance and x is the concentration used), using 5 mL of standard solutions and analyzing in triplicate. The results are expressed as % average value ± % RSD.

ASE water solubility: Solubility test of ASE was previously determined in distilled water as follows: an excess amount of extract (100 mg) was introduced into a flask containing 10 mL of water. According to the “shake flask method”, the sample was shaken for 3 days at room temperature (25 °C) [18]. After 3 days, the filtered supernatant solution was examined by UV-VIS apparatus at 280 nm (1 cm cell) to determine the amount of dissolved extract. Each analysis was made in triplicate.

ASE in vitro dissolution test: An aliquot of ASE samples corresponding to 3 mg of CA was carried out under sink conditions in 1000 mL of water using an SOTAX AT Smart Apparatus (Basel, CH), on line with a spectrophotometer at λ = 280 (UV/Vis spectrometer Lambda 25, Perkin-Elmer Instruments, MA, USA), and USP 37 dissolution test apparatus n.2: paddle, 100 rpm at 37 °C. All the dissolution/release tests were made in triplicate; only the mean values are reported in the graph (standard deviations <5%).

### 2.4. Formulation Studies

Phase-solubility studies: To determine the exact molar concentration of the solid CD-ASE complex and CD-CA complex chosen as reference, an excess amount of ASE (10,0 mg), expressed as CA (0.32 mg), or CD (0,32 mg), was suspended in 100 mL of water. Different amounts of CD (1:0, 1:1; 1:0.25; 0.5:1; 1:1; 1:2; 1:3; 1:4 CA/CD molar ratio for ASE:CD and 1:0, 0.5:1, 1:1; 1:1; 1:2; 1:3; 1:4 for CD-CA) were added. Then, samples were shaken and stored at 25 °C for 1 h and centrifuged (5 min at 3000 rpm). The supernatants were analysed in UV apparatus (1 cm cell; λ = 280 nm). The phase-solubility diagram was determined by the Higuchi and Connors method [19].

Complex preparation: The CD-ASE and CD-CA solid complexes were prepared with the freeze-dried method as follows: 8.125 mg of ASE, corresponding to 9.6 × 10^−3^ mol of CA or 1.25 × 10^−4^ mol of CA, were suspended in 10 or 1 mL of water, respectively, and then was added to 9.6 × 10^−3^ mol or 1.25 × 10^−4^ mol of CD, respectively. The sample was vortexed for 4 h, stored for 24 h at −4 °C, and lyophilized for 24 h to obtain the clathrate CD-ASE.

### 2.5. Complex Characterization

#### 2.5.1. Liophylization Process Yield (LPY), Actual Extract Content (AEC) and Inclusion Efficiency (IE)

The LPY is gravimetrically determined and expressed as the weight percentage of the final product compared to the total amount of the lyophilized materials.

The AEC was assessed by UV-VIS method as follow: 3 mg of CD-ASE or CD-CA were dissolved in 10 mL of deionized water, vortexed for 60 s and determined spectrophotometrically at **λ** 280 nm (1 cm cell; Spectracomp 602, Advanced Products SRL, Milan, Italy). Each analysis was made in triplicate and the results were expressed as average value. The IE was calculated from the ratio of AEC to theoretical extract content (TEC) in a freeze-dried complex according to the equation below.

IE (%) = (AEC/TEC) × 100(1)

#### 2.5.2. Absorption Spectra

The absorption spectra of 1:1 molar ratio complexes and raw materials were taken with a Spectracomp 602, Advanced Products SRL, Milan, Italy. The molar concentrations examined were 9.6 × 10^−3^ mol for CD and 9.6 × 10^−3^ mol for CA.

#### 2.5.3. Differential Scanning Calorimetry (DSC)

To study the thermal behavior on each sample, DSC was performed on an indium-calibrated Mettler Toledo DSC 822e (Columbus, OH, USA) as follows: ASE, CD, CD-CA and CD-ASE were exposed to one thermal cycle. The samples were placed in a pierced 40 μL aluminium pan and scanned (10 °C/min) between 25 and 350 °C. Melting temperature (Tm) and heats of fusion (DHm) were measured.

#### 2.5.4. Morphology

Samples were prepared by sprinkling a small amount of dry powder (10 g) onto a microscope slide and then were observed with a Zeiss Axiophot fluorescence microscope (FM) (Carl Zeiss Microscopy, Jena, Germany), with an optavar with a factor of 2.5× and a 40× 1.4 NA plan Apochromat oil immersion objectives (Carl Zeiss Vision, Müchen-Hallbergmoos, Germany) using standard 40,6-diamidino-2-phenylindole optics that adsorb violet radiation (max 372 nm) and emit a blue fluorescence (max 456 nm). Morphology and mean size of ASE pure, CD pure and CD-ASE complexes were also investigated by scanning electron microscopy (SEM) (Carl Zeiss EVO MA 10 microscope operating at 20 kV), after coating with Au/Pd. The average particle diameters were determined from an average of at least 20 observations.

#### 2.5.5. Solubility Studies: Complex Water Solubility (WS) and Dissolution Rate

WS analysis was conducted as previously reported for ASE water solubility according to the “shake flask method” (see Section 2.3). The in vitro dissolution/release tests of ASE from the complex were carried out under sink conditions (about 30 mg of extract corresponding to 10.25 mg of CA) in 1000 mL of water using a SOTAX AT Smart Apparatus (Sotax Group, Basel, CH, Switzerland), on line with a spectrophotometer at λ = 310 (UV/Vis spectrometer Lambda 25, Perkin-Elmer Instruments, Waltham, MA, USA), and USP 37 dissolution test apparatus n.2: paddle, 100 rpm at 37 °C. All the dissolution/release tests were made in triplicate; only the mean values are reported in the graph (standard deviations <5%).

### 2.6. Biological Activity

#### 2.6.1. Cell Culture

IEC-6 cells (CRL-1592; ATCC, Rockville, MD, USA) derive from normal rat intestinal crypt cells and were grown in Dulbecco’s modified Eagle’s medium (DMEM, 4 g/L glucose) with foetal bovine serum (FBS; 10% *v*/*v*), l-glutamine (2 mM), NaHCO_3_, (1.5 g/L) and bovine insulin (0.1 unit/mL). Cells used for these experiments were between the 16th and 19th passages.

#### 2.6.2. Cell Treatment

IEC-6 cells were plated and allowed to adhere for 24 h at 37 °C in a 5% CO_2_ atmosphere. ASE and formulation (CD-ASE; 50–5 μg/mL) were then added for 1 h and then co-exposed to lipopolysaccharides from *E. coli* (LPS; 10 μg/mL) plus interferon-γ (IFN; 10 U/mL) for different experimental times, as indicated below [20]. In order to further evaluate the antioxidant potential of almond ASE, and CD-ASE, in another set of experiments, the IEC-6 cells were incubated with the tested products (50–5 μg/mL) for 1 h alone, and then simultaneously to hydrogen peroxide (H_2_O_2_; 1 mM) [21].

#### 2.6.3. Antiproliferative Activity Evaluation

IEC-6 cells were plated on 96-well plates and, after adhesion, the medium was changed with either a new one alone or one containing ASE or CD-ASE (50–5 μg/mL) for 24 h. The antiproliferative activity was evaluated using the 3-(4,5-dimethylthiazol-2-yl)-2,5-diphenyltetrazolium bromide (MTT) [22]. MTT (5 mg/mL) was then added to the cells and after 3 h, 100 µL of a solution containing 50% (*v*/*v*) N,N-dimethylformamide, and 20% (*w*/*v*) sodium dodecyl sulphate (SDS; pH = 4.5) was added to the IEC-6 cells. The optical density (OD) of released formazan in each well was measured with a microplate spectrophotometer reader (Titertek Multiskan MCC/340-DASIT, Cornaredo, Milan, Italy) [23]. The antiproliferative activity was evaluated as % dead cells = 100 − ((OD treated/OD control) × 100).

#### 2.6.4. Tumor Necrosis Factor (TNF-α) Evaluation

TNF-α levels were assessed with an Enzyme-Linked Immuno Sorbent Assay (ELISA). IEC-6 cells were plated into 24-well plates (8.0 × 10^4^ cells/well) and, after 24 h, almond ASE and CD-ASE (50–5 μg/mL) were added. After 24 h, the medium was then collected and a commercial kit (e-Bioscience, San Diego, CA, USA) was used to perform the ELISA, according to the manufacturer’s instructions. Results were expressed as pg/mL, as previously reported [24].

#### 2.6.5. Evaluation of COX-2 and iNOS Expression by Cytofluorimetry

IEC-6 cells were plated into 96-well plates (2.0 × 10^3^ cells/well) and treated with almond ASE and CD-ASE for 24 h in LPS + IFN-stimulated IEC-6 cells. After the treatment with the tested products, IEC-6 cells were collected and washed with phosphate buffered saline (PBS), and then fixing solution was added to cells for 20 min. Cells were subsequently incubated in a fix perm solution for 30 min, and anti-COX-2 (BD Transduction Laboratories, Milan, Italy) and anti-iNOS (BD Transduction Laboratories, Milan, Italy) antibodies were then throw in for 1 h. Then, the secondary antibody, in fixing solution, was added to IEC-6 cells and a fluorescence-activated cell sorter (FACSscan; Becton Dickinson, Milan, Italy) and analyzed by Cell Quest software (version 4; Becton Dickinson, Milan, Italy) to evaluate the cell fluorescence [25].

#### 2.6.6. Intracellular Reactive Oxygen Species (ROS) Release Evaluation

The probe 2′,7′-dichlorofluorescein-diacetate (H_2_DCF-DA) was used to evaluate ROS intracellular production [26]. IEC-6 cells were plated in 24-well plates (8.0 × 10^4^ cells/well). After adhesion, almond ASE and CD-ASE (50–5 μg/mL) were added for 1 h alone and then simultaneously to LPS (10 μg/mL) plus IFN (10 U/mL) for 24 h. In other experiments, the cells were treated with the tested products, at the same concentrations, alone for 1 h and then simultaneously to H_2_O_2_ (1 mM) for another 1 h. IEC-6 cells were then collected, washed with PBS and incubated in PBS plus H_2_DCF-DA (10 μM). After an incubation of 15 min at 37 °C, cell fluorescence was evaluated using a fluorescence-activated cell sorter (FACSscan; Becton Dickinson, Franklin Lakes, NJ, USA), and was analyzed by Cell Quest software version 4 (Becton Dickinson, Milan, Italy) [27].

### 2.7. Data Analysis

Data are reported as mean ± standard error of the mean (s.e.m.) values of at least three different experiments each in triplicate. Statistical analysis was performed by analysis of variance test, and multiple comparisons were made by Bonferroni’s test by using Prism 5 (GraphPad Software, San Diego, CA, USA). *p*-values smaller than 0.05 were considered significant.

## 3. Results and Discussion

The Y ASE was 11.0 ± 0.05%. Considering that procyanidins are among the most abundant compounds in almond skins [11], (+) catechin was used as a marker and the ASE CA content was 32 ± 0.1%.

### 3.1. Technological Data

#### 3.1.1. Pre-Formulation and Formulation Studies

**Figure 1:**
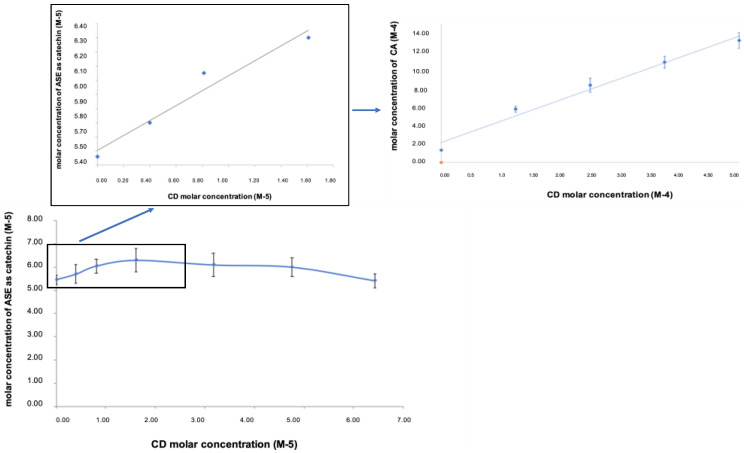
Solubility phase diagram of acetonic almond skins extract (ASE) in the presence of β-cyclodextrin (CD), compared to that of catechin (CA).

According to USP 37, ASE was considered practically insoluble in water (50.0 ± 1.5 mg/L) at room temperature, and the water dissolution test showed that no more than 45% of ASE dissolved in 45 min. These characteristics influence the in vivo active behavior, reducing its in vivo absorption and bioavailability [28]. For this reason, we considered CD as a material to improve ASE solubility and to enhance the dissolution rate [8]. In CD-ASE, the ascendant portion of the phase solubility diagram (Figure 1) was similar to that for (+) catechin pure (A_L_ Type) [15], while the total profile conducted to evaluate the CD amount required showed a Bs-type profile [19]. These results were because ASE is a synergy of actives, each with a different stereochemistry and interaction force. Taken singularly, the actives could present a different profile, like (+) catechin, and could be also enclosed totally in CD cavity [29]. A 1:1 stoichiometry complex CD-ASE was found by the trend of the ascending portion that increases linearly below the 1:2 ASE/CD molar ratio. The following short plateau indicated that, at higher CD concentrations, an insoluble product or a different stoichiometry complex in the solution was formed. For this reason, conventionally, we assumed a 1:1 ASE:CD molar ratio to obtain an improvement in water solubility (from 50.0 ± 1.5 mg/L to 185 ± 1.1 mg/L) and an enhancement of the dissolution rate from 13% to 90%, at just 15 min. Moreover, a complete ASE dissolution (100%) occurred in 45 min.

#### 3.1.2. Complex Characterization

LPY was 100.0 ± 0.2. Considering the ASE amount in the CD-ASE complex, TEC expressed as CA equivalent was 16.0 ± 0.5%, and AEC resulted 14.0 ± 1.0% with an IE value of 88.0 ± 2.0%.

**Figure 2:**
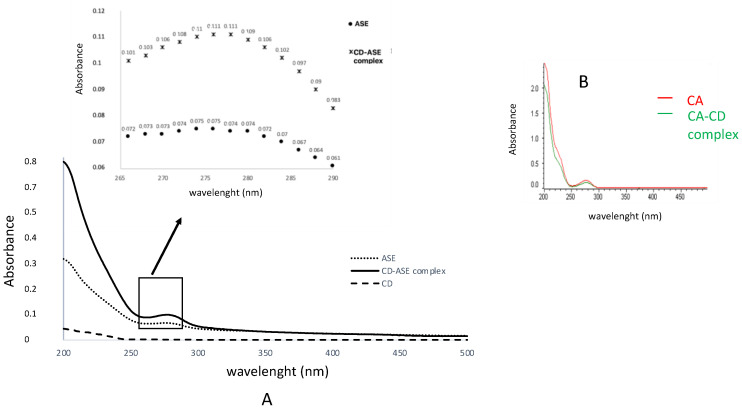
UV spectra analyses of: (**A**) ASE-CD complex in comparison with ASE and CD pure materials; (**B**) CD-CA complex in comparison with CA pure.

The interactions of CD-ASE in the aqueous solution were examined by comparing the UV-Vis spectra of ASE with those of CD and the CD-ASE complex (Figure 2A). In CD-ASE, hypsochromic and hyperchromic effects were observed. In fact, ASE intense maximum peak (280 nm), decrease in the CD-ASE complex (275 nm) with a higher intensity of k band. These results were probably due to a partial inclusion of ASE in CD cavity with weak bonds [30], also suggesting H bond formation.

**Figure 3:**
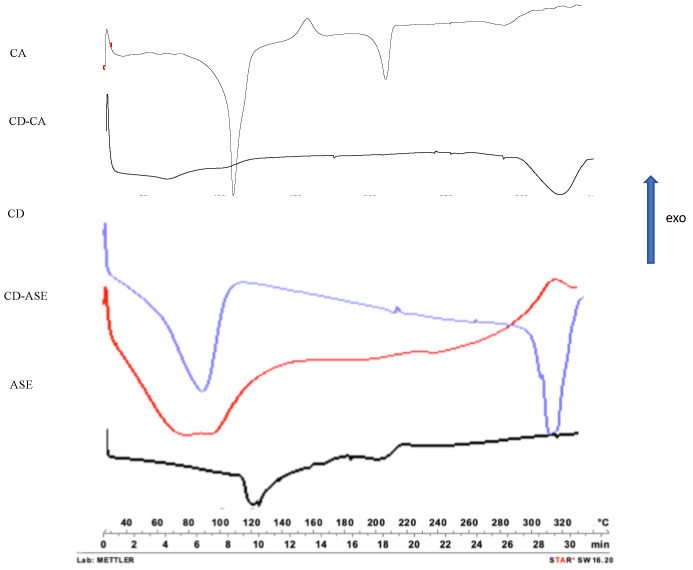
Differential scanning calorimetry of pure materials (ASE, CD, CA), CD-ASE and CD-CA complex.

Differently, the CD-CA complex evidenced a hypochromic effect, probably due to the complete inclusion of CA in the CD cavity. These results were confirmed by DSC, FM and SEM analyses. The CD-ASE thermogram (Figure 3) shows a series of ASE and CD characteristic peaks from 70 to 350 °C with slight shifts and minor intensity, confirming that a part of ASE was included in cyclodextrin cavity. Moreover, in a complex thermogram, the peak fusion of the crystalline structure of CD disappears and no ASE degradation peak was observed, confirming a physical interaction from ASE and CD that is also able to protect ASE from high degradation temperatures. Instead, the CD-CA thermogram was similar to that CD, and CA peaks disappeared probably due to the incorporation of all the guest molecules into the CD cavity.

**Figure 4:**
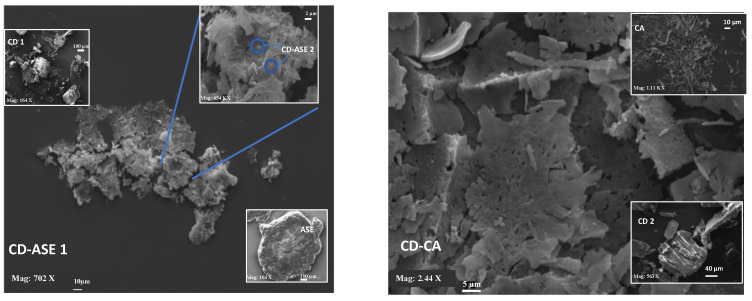
SEM micrographs at different magnifications (Mag) (CD-ASE 1, 702 X; CD-CA, 2.44 KX; CD 1, 164 X; CD 2, 563 X; ASE, 164 X; CD-ASE 2, 654 KX; CA, 1.11 KX; frame average, *N* = 1)**.**

SEM micrographs (Figure 4) showed an amorphous structure (about 500 μ), of unformulated ASE, while CD presents crystalline structures with an irregular rectangular structure of a different size. In CD-ASE, a partial change was possibly noted in the CD structure. In fact, tiny CD structures (2–5 μ) and a few ASE amorphous materials (0.5–1 μ) were observed on their surface. Aggregates were also shown. Instead, in CD-CA, the original morphology of both components disappeared. FM analyses were carried out only for CD-ASE, because of the major difficulties identifying ASE in the SEM of the complex.

**Figure 5:**
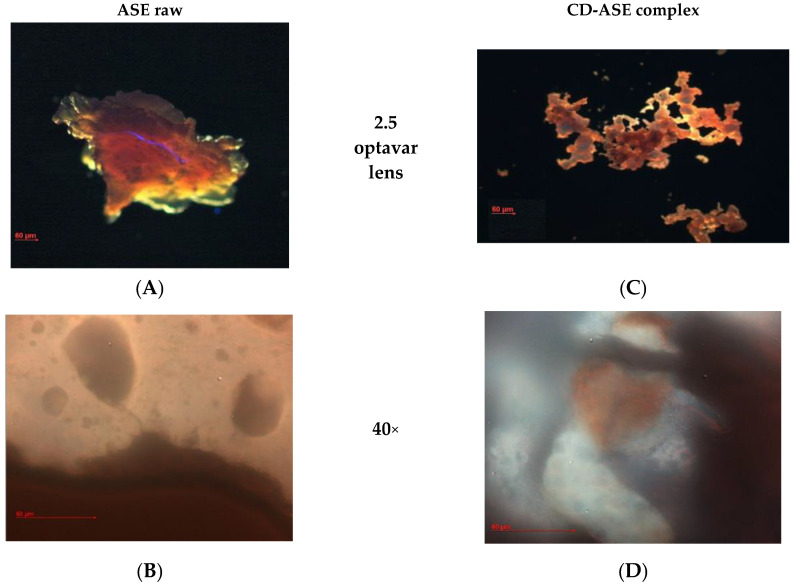
Fluorescence (FM, optical 2.5 and ×40) microphotographs of ASE raw (**A**,**B**) and ASE-CD complex (**C**,**D**).

In the FM microphotograph (Figure 5), the type of ASE complexation was more visible. CD-ASE presents (Figure 5C,D) a CD cavity (spherical shadow areas) in which ASE (Figure 5A,B) was only partially embedded, while another part is located on the CD surface, confirming the SEM results.

#### 3.1.3. CD-ASE Solubility Studies

**Figure 6:**
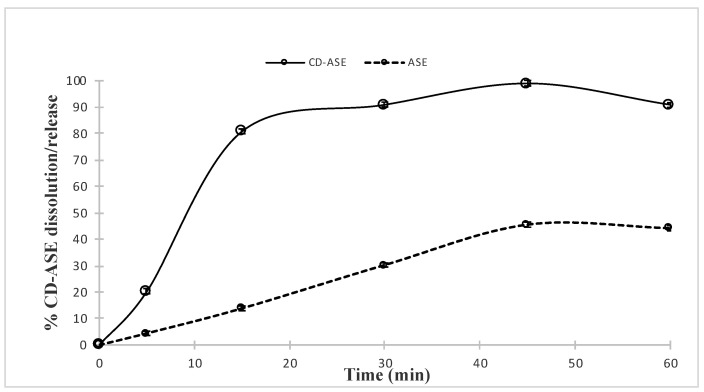
Dissolution/release profile of CD-ASE complex in comparison with ASE raw dissolution profiles in water.

CD was able to improve water ASE solubility and enhance its dissolution rate. Indeed, the solubility increased from 50 to 183 mg/L and about 90% of ASE from CD-ASE was released and dissolved in 15 min, reaching 100% in 45 min, with respect to pure ASE that dissolved only 13% and 45%, respectively, in the same times (Figure 6).

### 3.2. Biological Activity

#### 3.2.1. ASE, and CD-ASE Did Not Affect IEC-6 Viability

To elucidate the influence of ASE, and CD-ASE on IEC-6 viability in our experimental conditions, cells were treated with the products (50–5 μg/mL) for 24 h. Our data indicated that ASE and CD-ASE did not significantly affect IEC-6 viability (ASE 50 μg/mL 9.58 ± 1.15; ASE 10 μg/mL 9.03 ± 1.45; ASE 5 μg/mL 0.00 ± 0.00; CD-ASE 50 μg/mL 1.00 ± 0.58; CD-ASE 10 μg/mL 0.67 ± 0.00; CD-ASE 5 μg/mL 0.00 ± 0.00).

#### 3.2.2. ASE and CD-ASE Reduced TNF-α Release in LPS + IFN-Stimulated IEC-6 Cells

**Figure 7:**
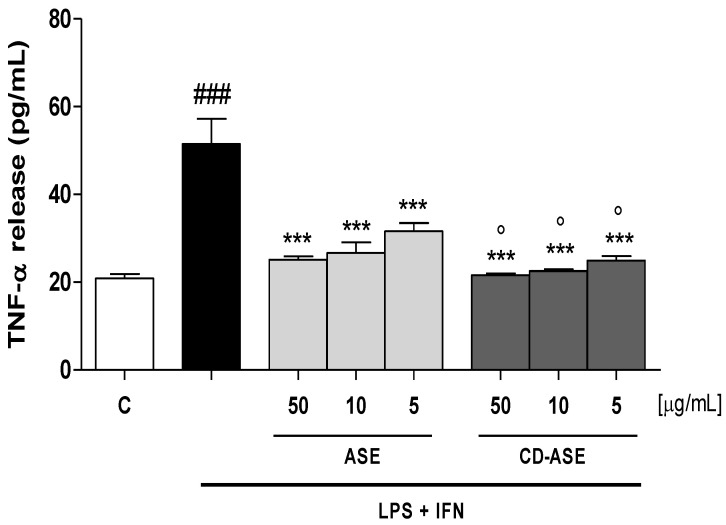
Effect of acetonic almond skins extract (ASE), and formulation (CD-ASE; 50–5 µg/mL) on TNF-α release, induced by LPS + IFN in IEC-6 cellular medium, evaluated by ELISA assay. Data are expressed as pg/mL of TNF-α release. *** denotes *p* < 0.001 vs. LPS + IFN; ° denotes *p* < 0.05 vs. ASE; ### denotes *p* < 0.001 vs. control (C).

TNF-α is one of the main cytokines regulating the inflammatory response at the intestinal level [31]. The effect of ASE and CD-ASE on TNF-α levels in IEC-6 cellular medium was evaluated using an enzyme-linked immunosorbent assay (ELISA). Our results showed that ASE and CD-ASE (50–5 μg/mL) significantly inhibited TNF-α release, induced by LPS + IFN, in IEC-6 cells at all tested concentrations (*p* < 0.001 vs. LPS + IFN; Figure 7). Interestingly, we can observe that CD-ASE reduced TNF-α release in IEC-6 significantly with respect to ASE alone (*p* < 0.05 vs. ASE). A significant inhibition effect on TNF-α of ASE-CD was observed with respect to ASE alone, which could be due to the different effects of cyclodextrins on biological barriers. In particular, the enhancement of active solubility and drug release improve the in vivo absorption and bioavailability. The improvement in permeability is also an important effect that allows a major distribution of active ingredients [32]. The ASE-CD effect observed here is in accordance with other studies in various models of inflammation, also at intestinal level [33,34,35].

#### 3.2.3. ASE and CD-ASE Inhibited COX-2 and iNOS Expression in LPS + IFN-Stimulated IEC-6 Cells

**Figure 8:**
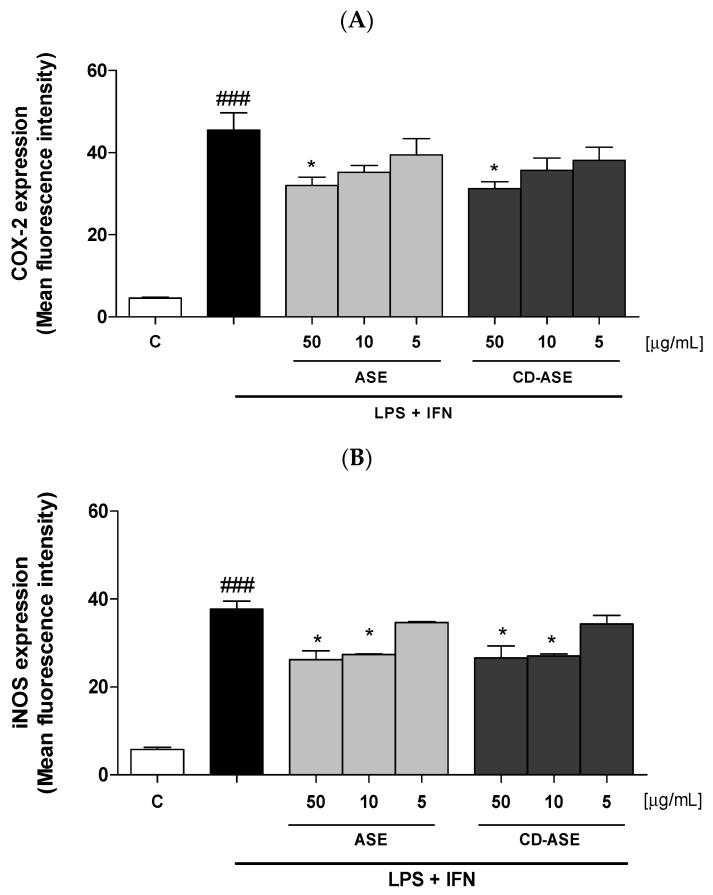
Effect of Acetonic almond skins extract (ASE), and formulation (CD-ASE; 50–5 µg/mL) on COX-2 (**A**) and iNOS (**B**) expression, induced by LPS + IFN in IEC-6 cells, evaluated by the cytofluorimetric technique. * denotes *p* < 0.05 vs. LPS + IFN; ### denotes *p* < 0.001 vs. control (C).

In addition to TNF-α, proinflammatory enzymes, such as COX-2 and iNOS, are also elevated as a consequence of the intestinal inflammation. In particular, COX-2 and iNOS expression contribute both to the amplification of the inflammatory response (via TNF-α) and to oxidative stress [36,37]. In order to assess their expression profile in IEC-6, cells were treated with ASE and CD-ASE (50–5 µg/mL) in inflammatory conditions. After 24 h, we observed a significant decrease in COX-2 (*p* < 0.05 vs. LPS + IFN; Figure 8A) and iNOS (*p* < 0.05 vs. LPS + IFN; Figure 8B) expression. In particular, COX-2 was inhibited by ASE and CD-ASE at the highest concentration. Concerning iNOS expression, IEC-6 was significantly inhibited by ASE and CD-ASE at the two highest concentrations.

#### 3.2.4. ASE and CD-ASE Reduced Intracellular ROS Release in IEC-6 Cells

Oxidative stress is a condition able to affect the organism at various levels and to significantly contribute to the pathogenesis of many disease [38,39,40,41]

**Figure 9:**
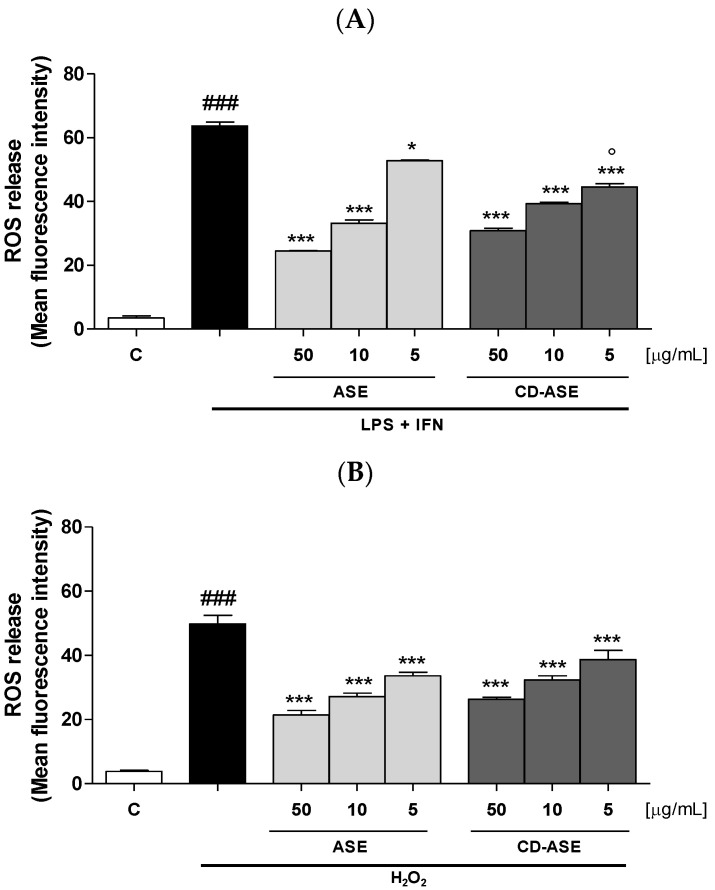
Effect of Acetonic almond skins extract (ASE), and formulation (CD-ASE; 50–5 µg/mL) on intracellular ROS release, in LPS + IFN (**A**) and H_2_O_2_ (**B**)-IEC-6 treated cells, detected by the probe of 2′,7′ dichlorofluorescein-diacetate (H_2_DCF-DA). Data are expressed as mean ± SEM of mean fluorescence intensity. *** and * denote respectively *p* < 0.001 and *p* < 0.01 vs. LPS + IFN or vs. H_2_O_2_; ° denotes *p* < 0.05 vs. ASE; ### denotes *p* < 0.001 vs. control (C).

Inflammatory response is denoted by elevated levels of highly reactive intermediates as ROS which, in turn, are able to promote the inflammatory cascade acting on transcription factors and cytokine production [36,42]. Thus, the control of ROS is of primary importance in the treatment of intestinal inflammation. Our results indicated that that the tested compounds (50–5 μg/mL) significantly inhibited ROS production in IEC-6 cells (*p* < 0.05 vs. LPS + IFN; Figure 9A) at all the sample tested concentrations. In particular, CD-ASE significantly inhibited ROS release compared to ASE alone (*p* < 0.05), but only at the concentration of 5 µg/mL, thus indicating that the complex does not, overall, show superior efficacy to the ASE alone. To further evaluate the antioxidant potential of the studied products, the inhibition of ROS release by ASE and CD-ASE (50–5 μg/mL) was also evaluated in IEC-6 cells treated with a pro-oxidant stimulus given by H_2_O_2_ (1 mM). In these experimental conditions, as assessed during inflammation, the tested compounds exhibited a significant antioxidant activity by inhibiting ROS release at all the tested concentrations (*p* < 0.001 vs. H_2_O_2_; Figure 9B).

## 4. Conclusions

Sicilian almond skin acetonic extract contains polyphenols, well-known for their antioxidant activity [10,11,12,13,14,15,16,17]. Unfortunately, it was characterized by a low water solubility and a consequent slow and incomplete in vitro dissolution rate that affects in vivo bioavailability. These characteristics also can influence the pharmacological action, reducing the effectiveness of the product.

The technological approach used, which involved the complexation of the extract with beta-CD (GRAS list) and subsequent lyophilization, was optimal for the proposed objectives, increasing ASE solubility and dissolution rate, and also allowing one to obtain the production yields and efficiencies of satisfactory complexation. Furthermore, the anti-inflammatory evaluation of the pure and formulated extract supports their potential application in intestinal inflammation conditions. In particular, this potential is highlighted by the significant efficacy of the formulated extract on TNF-α inhibition, one of the main mediators of intestinal inflammation. Therefore, this approach seems to be useful to obtain an almond-based system with improved technological characteristics in the extract and able to use it as a dietary supplement in health products, obtaining a product with a high value-added, with a reduction also in the industrial almond waste disposal costs.
